# High-grade childhood intra-parenchymal brain tumor clustering with ATRT and expanding the cancer spectrum related to inherited SMARCE1 truncating variations

**DOI:** 10.1186/s40478-022-01325-8

**Published:** 2022-02-14

**Authors:** Fabien Forest, Julien Masliah-Planchon, Claire Berger, Fabienne Prieur, Elodie Girard, Fanny Burel-Vandenbos, Claire Boutet, François Vassal, Franck Bourdeaut, Catherine Godfraind

**Affiliations:** 1 Departments of Pathology and Molecular Biology of Tumors, University Hospital of Saint Etienne, Saint Etienne, France; 2 Department of Genetic, Curie Institute, Paris, France; 3 Department of Pediatric Haemato-Oncology, University Hospital of Saint Etienne, Saint-Étienne, Lyon University, Jean Monnet University, INSERM, U1059, Sainbiose, Saint Etienne, France; 4 Department of Genetic, University Hospital of Saint Etienne, Saint Etienne, France; 5 Department of Pathology, Université Côte d’Azur, CHU Nice, Nice, France; 6 Department of Radiology, University Hospital of Saint Etienne, Saint Etienne, France; 7 Department of Neurosurgery, University Hospital of Saint Etienne, Saint Etienne, France; 8 SIREDO Oncology Center (Care, Innovation and Research for Children and AYA With Cancer), PSL Research University, Institut Curie, Paris, France; 9 UF of Neuropathology, Clermont-Ferrand CHU and UMR Inserm/Université d’Auvergne U1071, Clermont-Ferrand, France; 10 CHU de Saint Etienne. Hôpital Nord. Service d’Anatomie et Cytologie Pathologiques, Avenue Albert Raimond, 42055 Saint Etienne, Cedex2, France

## Letter to the Editor

SMARCE1 (SWI/SNF related, matrix associated, actin dependent regulator of chromatin, subfamily e, member 1) is a constant component of the SWI/SNF (SWItch/Sucrose Non-Fermentable) chromatin remodeling complexes. While heterozygous missense variants are responsible for the rare genotype of Coffin-Siris syndrome, heterozygous truncating variants predispose individuals to an increased risk of clear cell meningiomas (CCM) that occur from childhood to adulthood. Here we report an undescribed supra-tentorial intra-parenchymal malignant tumor with an inherited heterozygous truncating variant of *SMARCE1,* and thereby expand the spectrum of SMARCE1-related cancer predisposition syndrome.

A 4-year-old child without congenital abnormality and normal development was addressed for seizures. MRI revealed a 40 × 33 × 30 mm right temporal tumor. There was no family history of tumor, especially no brain tumor or meningioma. The tumor was not dura matter based on imaging and on per-operative findings. Complete resection was performed. The patient was treated by radiotherapy and temozolomide. After 30 months of follow-up the tumor did not relapse.

Histopathological findings showed a highly cellular tumor devoid of specific pattern. Tumor cells were small to medium sized with moderate amount of cytoplasm. There was no clear cell morphology neither rhabdoid cells. More than 20 mitoses/2mm^2^, endothelial proliferation and necrosis were observed. Automated immunohistochemistry demonstrated labeling of tumor cells by Olig2 (AF2418, R1D Systems) stained most of tumor cells, GFAP (6F2, Dako), synaptophysin (DAK-SYNAP, Dako), EMA (E29, Dako) in 10% of tumor cells, alpha smooth actin (1A4, Dako) stained most of tumor cells, and p53 (DO-7, Dako) diffusely stained tumor cells, Vimentin (V9, Dako) diffusely stained tumor cells. There was no staining by SSTR2A (ab134152, Abcam), IDH1^R132H^ (H09, Dianova), H3K27M (K27M, Sigma-Aldrich), LIN28(A177, Cell Signaling), NFKappaB (D14E12, Cell Signaling), Internexine Alpha (BB300-140, Novus), AE1AE3 (AE1/AE3, Dako-Agilent), FGFR3 (SC-13121, Santa Cruz), CD34 (QBEnd 10, Beckman Coulter) antibodies. There was no loss of ATRX (poly, Sigma-Aldrich) H3K27me3 (H3K27Me3, Sigma-Aldrich), BAF47 (25/BAF47, BD Biosciences), BRG1 (ab110641, Abcam). Ki67 (MIB1, Dako) stained 80% of tumor cells.

Given the absence of definite diagnosis, we next investigated whether molecular characterization could help classify the tumor. Methylation profiling (Illumina EPIC Human Methylation microarray) failed to classify the tumor in any known subgroup: the DNA methylation classifier V11b4 gave as prediction class “atypical teratoid and rhabdoid tumor (ATRT)” with a low calibration score of 0.67. Its latest version V12.3 assessed the lesion to “GBM pedMYCN” with a score of 0.17 while “ATRT-SHH subtype” had a score of 0.13. On a t-SNE plot perfomed on v11b4 version of the DNA methylation classifier, the tumor was closely related to ATRT, and clearly distinct from clear cell meningioma.

Then, a targeted NGS custom panel of 571 genes involved in oncogenesis was performed on the tumor DNA; it surprisingly revealed a c.460G > T / p.(Glu154*) pathogenic variation (PV) in *SMARCE1*. The inactivation of *SMARCE1* was confirmed at the protein level with anti-BAF57 (poly, Sigma-Aldrich)*.* No other variant was found in the SWI/SNF complex genes; noticeably*,* NGS also revealed a c.1009C > T / p.(Arg337Cys) PV in *TP53.* The final retained diagnosis was a malignant tumor with *SMARCE1* inactivation, unclassifiable in the current World Health Organization Classification.

The chromosome CNV profile showed a complex profile unlikely found in ATRT or clear cell meningioma. It also revealed an isodisomy of the whole chromosome 17 where *SMARCE1* and TP53 genes are located. Besides, the relatively complex genomic profile and the mutation in *TP53* are not consistent with typical ATRT since those tumors most generally harbor a very simple profile and no PV except *SMARCB1* or *SMARCA4* alterations. Targeted mRNA sequencing (FusionPLex Comprehensive Panel, ArcherDx) on formalin-fixed, paraffin embedded material did not show any fusion gene.

The presence of a *SMARCE1* biallelic PV in a brain tumor occurring at 4 years prompted us to test the germline DNA, which confirmed that the child was bearing the heterozygous PV in the lymphocytes. The familial screening revealed that the PV was inherited from the child’s unaffected father (54 years old at diagnosis).

**Fig. 1:**
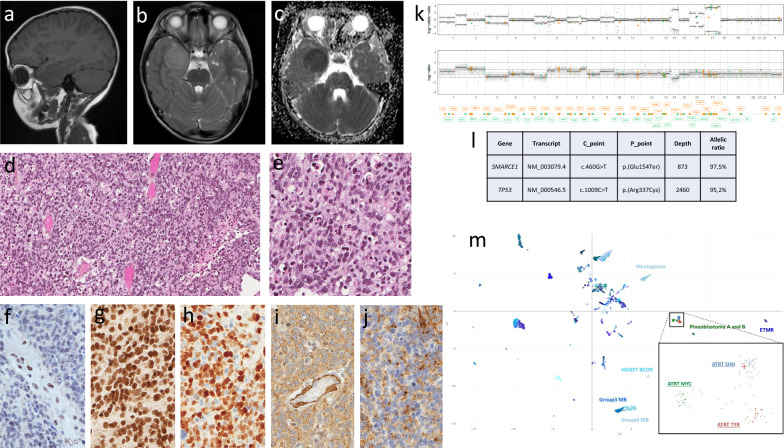
**a** MRI sagittal plane, T1 sequence without gadolinium injection showing a hypointense right temporal tumor. **b** MRI axial plane, T2 sequence showing a hyperintense right temporal tumor. **c** MRI axial plane, apparent diffusion coefficient (ADC) values showing a low ADC in the right temporal tumor. **d**, **e** Hematoxylin and Eosin (H&E) stain × 200 (**d**) and × 400 (**e**) showing a hypercellular tumor with pleomorphic cells harboring numerous mitoses. **f** SMARCE1 immunohistochemistry, × 400, showing a lack of expression in tumor cells with positive cells in a vessel as an internal control. **g** BAF47 immunohistochemistry, × 400, showing a maintained nuclear expression in tumor cells. **h** olig2 immunohistochemistry, × 400, showing a diffuse nuclear expression in tumor cells. **i** α-smooth actin immunohistochemistry, × 400, showing a cytoplasmic expression in tumor cells. **j** EMA immunohistochemistry, × 400, showing a patchy expression in tumor cells. **k** Copy number variation profile showing a complex profile. **l** Details of *SMARCE1* and *TP53* pathogenic variants. **m** UMAP showing the close localization of the tumor with ATRT

Truncating germline variants of *SMARCE1* are so far restrictedly associated to CCM inferring a central role of this gene to CCM oncogenesis [4]. To the best of our knowledge, the only other brain tumor reported in the context of *SMARCE1* PV was an anaplastic astrocytoma occurring in a child with a Coffin-Siris syndrome and demonstrating a missense heterozygous PV [3]. The biallelic hit resulting in the loss of protein expression [5] further argues in favor of a driver role of SMARCE1 in our case. Thus, the tumor we report enlarges the spectrum of SMARCE1-related brain tumors to a so far undescribed high-grade neoplasm. Indeed, a CCM could be formally ruled out because the tumor was not dura matter based, microscopic findings are not consistent with clear cell meningioma, immunohistochemical features were those of a tumor without SSTR2A expression and methylation profiling was clearly in disfavor of this diagnosis. Furthermore complex CNV profile was found whereas recurrent chromosomal aberrations in CCM are chromosome 17q (segmental) loss, chromosome 6q loss and chromosome 22q loss. Interestingly, the methylation profiling was the most related to ATRT, which may indicate some kindship with this family of tumors due to SWI/SNF deficiency [2]. Besides, the relatively complex genomic profile and the mutation in *TP53* are not consistent with typical ATRT, those tumors most generally harboring a very simple profile and no PV other than *SMARCB1* or *SMARCA4* alterations. Nevertheless, given the association of mutations in SWI/SNF proteins with ATRT and the close clustering of the tumor with ATRTs in methylation analysis, it is not possible to formally exclude that this tumor could be an ATRT with a SMARCE1 mutation (Fig. 1).

Altogether, this case expands the spectrum of SWI/SNF altered related tumors and of SMARCE1-related predisposition syndrome. Whether this risk should impact genetic counseling and tumor surveillance will depend on potential further reports of similar findings in individuals with *SMARCE1* germline truncating variants.

## Data Availability

The datasets used and/or analysed during the current study available from the corresponding author on reasonable request.
